# Hybrid α-Fe_2_O_3_@Ni(OH)_2_ nanosheet composite for high-rate-performance supercapacitor electrode

**DOI:** 10.1038/srep31751

**Published:** 2016-08-24

**Authors:** Hong Jiang, Haifeng Ma, Ying Jin, Lanfang Wang, Feng Gao, Qingyi Lu

**Affiliations:** 1 Department of Materials Science and Engineering, National Laboratory of Solid State Microstructures, Collaborative Innovation Center of Advanced Microstructures, Nanjing University, Nanjing 210093, P. R. China; 2 State Key Laboratory of Coordination Chemistry, Coordination Chemistry Institute, School of Chemistry and Chemical Engineering, Collaborative Innovation Center of Advanced Microstructures, Nanjing National Laboratory of Microstructures, Nanjing University, Nanjing 210093, P. R. China; 3 College of Biological and Chemical Engineering, Anhui Polytechnic University, Wuhu 241000, Anhui P. R. China

## Abstract

In this study, we report a facile fabrication of ultrathin two-dimensional (2D) nanosheet hybrid composite, α-Fe_2_O_3_ nanosheet@Ni(OH)_2_ nanosheet, by a two-step hydrothermal method to achieve high specific capacitance and good stability performance at high charging/discharging rates when serving as electrode material of supercapacitors. The α-Fe_2_O_3_@Ni(OH)_2_ hybrid electrode not only has a smooth decrease of the specific capacitance with increasing current density, compared with the sharp decline of single component of Ni(OH)_2_ electrode, but also presents excellent rate capability with a specific capacitance of 356 F/g at a current density of 16 A/g and excellent cycling stability (a capacity retention of 93.3% after 500 cycles), which are superior to the performances of Ni(OH)_2_ with a lower specific capacitance of 132 F/g and a lower capacity retention of 81.8% at 16 A/g. The results indicate such hybrid structure would be promising as excellent electrode material for good performances at high current densities in the future.

Energy crisis caused by the increasing consumption of fossil fuels calls for urgent development of flexible energy storage and conversion devices. Supercapacitor, also known as electrochemical capacitor (EC), with low cost, safety, long cycling life, fast charging/discharging rates and high power density, is considered to be a promising new star as energy storage devices^1^,^2^. They can take over batteries in electrical energy storage when meeting with short-term power boosts, such as emergency power supplies and peak power assistance in electrical equipment^3^,^4^. However, compared to rechargeable batteries, the critical defect of lower energy density still hinders EC’s practical applications. It is highly desirable for advanced supercapacitors to have higher operating voltage, higher energy/power density and longer cycle life to meet the energy demands for practical applications in the future^5^,^6^. At the same time, with the increasing demand for fast charge/discharge performance of energy storage device, it is imperative for scientists to improve the performance of all aspects of supercapacitor at high current densities, including specific capacitance (C), energy density (E) and stability. Energy density can be improved by maximizing the specific capacitance and/or the cell voltage (V) according to the following equation:^5^,^7^,^8^ *E* = 0.5*CV *2. To increase the energy density of capacitors, pseudocapacitors, which differ from normal double-layer capacitors in energy storage, have been extensively developed to increase the operation voltage by drawing on the theory of the Faradic electrode of Li-ion batteries^9^. Therefore, pseudocapacitive materials with high specific capacitance and excellent stability at high current densities emerge as the times require.

It is generally accepted that the performance of the capacitors depends greatly on their electrode materials. Recently, abundant pseudocapacitive materials including oxides^10^,^11^,^12^, hydroxides^13^,^14^ and polymers^15^,^16^ have been explored for excellent energy storage performances. Although these hydroxides or oxides have large theoretical specific capacitance and good performances in short terms, such “pseudocapacitors” often bend their knees to rate capability and reversibility during long-term Faradic redox reactions^4^,^17^. Nanomaterials have attracted great interest in energy conversion and storage devices because of their unique electrical properties^18^. Among them, two dimensional (2D) nanomaterials, whose thickness is just several atomic layers, become the brightest stars for their high specific surface areas and abundant surface electrochemical active sites^19^. Yet, single component of 2D nanomaterials, like Ni(OH)_2_ nanosheets, may suffer capacitance decrease after many cycles at high current densities, which prevents Ni(OH)_2_ from being advanced electrode material. Fortunately, scientists have dedicated numerous efforts to solve the problems and achieved great successes. It is a great approach to combine two individual constituents for better electrochemical performances. For example, Ni(OH)_2_/Graphene composite^4^,^5^, Co_3_O_4_/3D Graphene^20^, MnO_2_@NiO^21^, and 3D hybrid nanostructure Ni(OH)_2_@Fe_2_O_3_^18^, have significantly enhanced the capacitance and stability performance at high current densities. However, the hybrid composite consisted of two kinds of 2D inorganic graphene-like materials has not been reported.

Herein, for the first time, we presented a facile route to synthesize α-Fe_2_O_3_ nanosheet@Ni(OH)_2_ nanosheet hybrid for electrode material of supercapacitor. This distinctive structure has the following advantages. Firstly, α-Fe_2_O_3_ and Ni(OH)_2_ are both 2D nanosheets whose 2D structures can bring controllable electrical properties and high specific surface areas for superior electrochemical activities^19^,^22^,^23^. Secondly, the introduction of α**-**Fe_2_O_3_ can reduce the aggregation of individual Ni(OH)_2_ nanosheets into larger assemblies^17^,^24^,^25^. Thirdly, by hybriding 2D Ni(OH)_2_ with α-Fe_2_O_3_, synergetic effect is expected to give both high rate capability and good recyclability. The as-prepared α-Fe_2_O_3_@Ni(OH)_2_ nanosheet hybrid exhibits excellent rate capability with a specific capacitance of 356 F/g at a current density of 16 A/g and excellent cycling stability (a capacity retention of 93.3% after 500 circles), which are superior to the performances of Ni(OH)_2_ with a specific capacitance of 132 F/g and a capacity retention of 81.8%.

## Results and Discussions

**Figure 1:**
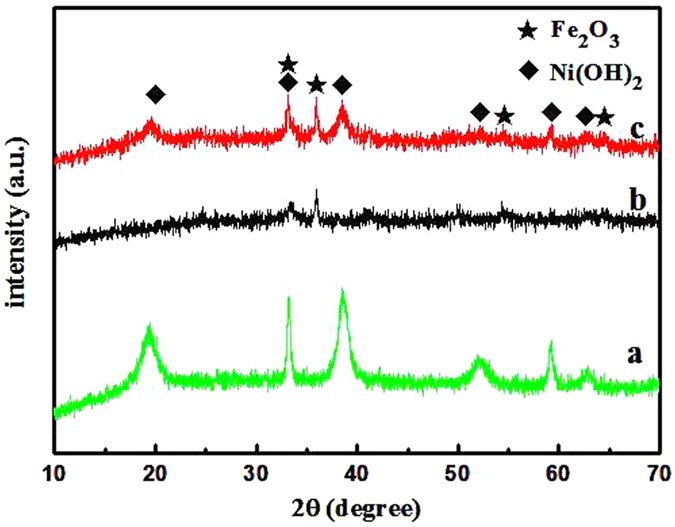
XRD patterns of (**a**) Ni(OH)_2_ nanosheets; (**b**) α-Fe_2_O_3_ nanosheets and (**c**) α-Fe_2_O_3_@Ni(OH)_2_ nanosheet hybrids.

**Figure 2:**
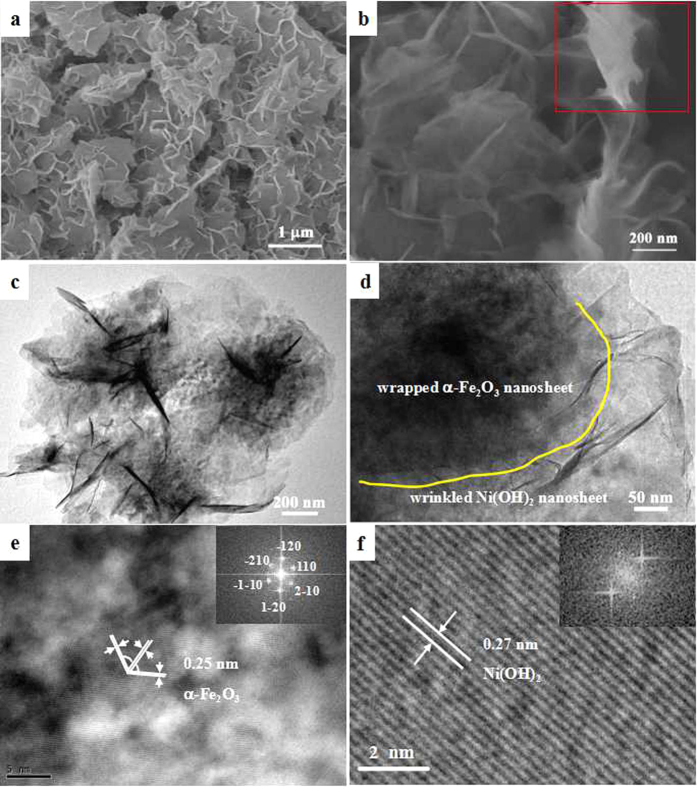
(**a**,**b**) SEM images and (**c**,**d**) TEM images and (e,f) HRTEM images of α-Fe_2_O_3_@Ni(OH)_2_ nanosheet hybrids.

The α-Fe_2_O_3_ nanosheet@Ni(OH)_2_ nanosheet hybrid was fabricated through a two-step hydrothermal route. First, ultrathin α-Fe_2_O_3_ nanosheets were synthesized by a metal ion intervened hydrothermal method with Al^3+^ as structure director according to our previous report^26^, then Ni(OH)_2_ nanosheets was produced onto the surface of as-prepared α-Fe_2_O_3_ with carboxymethylcellulose (CMC) as structure director. For comparison, the same procedure was also applied to prepare Ni(OH)_2_ without the addition of α-Fe_2_O_3_. Typical X-ray diffraction (XRD) patterns of Ni(OH)_2_, α-Fe_2_O_3_ and α-Fe_2_O_3_@Ni(OH)_2_ hybrid are shown in Fig. 1. In Fig. 1a, all of the diffraction peaks can be indexed to hexagonal Ni(OH)_2_ (JCPDS No. 03-0177), confirming the formation of single Ni(OH)_2_ nanocrystals with high purity. All the diffraction peaks in Fig. 1b can be indexed to hematite α-Fe_2_O_3_ (JCPDS No. 33-0664) and no obvious impurity peaks can be observed, indicating the high purity of the product with the addition of Al^3+^. After introducing Ni(OH)_2_ onto the surface of α-Fe_2_O_3_, both diffraction peaks of α-Fe_2_O_3_ and Ni(OH)_2_ emerge as shown in Fig. 1c, suggesting the formation of the composite composed of α-Fe_2_O_3_ and Ni(OH)_2_. Figure S1 displays scanning electron microscopy (SEM) images of two single materials. As shown in Figure S1a, the obtained α-Fe_2_O_3_ is composed of monodisperse nanosheets with 2D size of 500 nm and thickness of about 2 nm. Figure S1b demonstrates that under the hydrothermal conditions without the addition of α-Fe_2_O_3_, the obtained Ni(OH)_2_ crystals have flower-like structures assembled by nanosheets. Figure 2 shows SEM, transmission electron microscopy (TEM) and high-resolution TEM (HRTEM) images of α-Fe_2_O_3_@Ni(OH)_2_ nanosheet hybrid. From the SEM images shown in Fig. 2a,b, it is clearly observed that the hybrid presents a sandwich-like structure with wrapped α-Fe_2_O_3_ serving as intermediate layer and attached Ni(OH)_2_ nanosheet as coating layers. The typical structure can be clearly observed in red rectangle of Fig. 2b. The α-Fe_2_O_3_ nanosheets are tightly covered by Ni(OH)_2_ nanosheets with a lot of folds. TEM images shown in Fig. 2c,d also show that the center part of the structure is much darker than the edges confirming the sandwich-like structure of the nanosheet hybrid. Central part of the image contains α-Fe_2_O_3_ nanosheet covered by Ni(OH)_2_ nanosheets. Single wrinkled Ni(OH)_2_ nanosheet occupies most parts of edge section. Further evidences can be obtained from HRTEM characterizations. Figure 2e,f show HRTEM images of different parts of the hybrids. As shown in Fig. 2e captured from central part of the TEM image, three directions of fringes with same spacings of 0.25 nm and the corresponding fast Fourier transform (FFT) in inset of Fig. 2e indicate that they belong to hexagonal phase of α-Fe_2_O_3_ (PDF: 33-0664). At the same time, HRTEM image in Fig. 2f shows perfect aligned crystal lattice planes from outer edge, corresponding to {100} planes of Ni(OH)_2,_ whose interplanar spacing is 0.27 nm. The HRTEM images presents the changes of 2D lattice fringe from inside to outside, directly proving the structure characteristic of hybrid.

**Figure 3:**
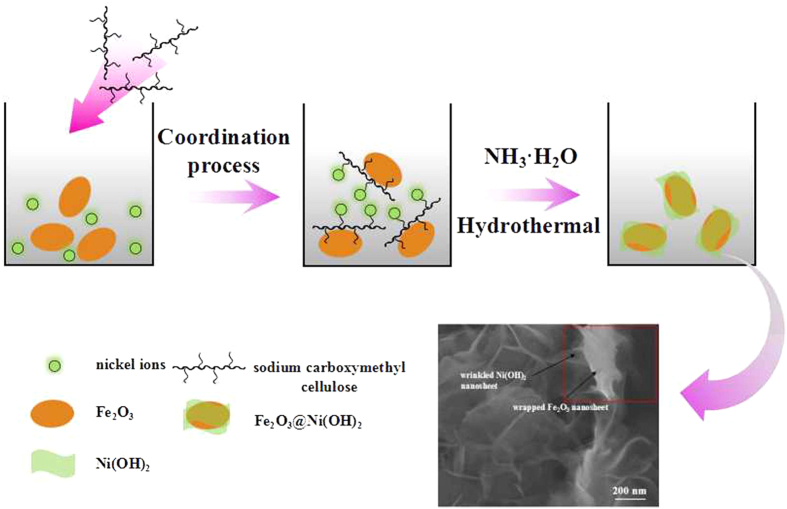
Schematic synthesis process of α-Fe_2_O_3_@Ni(OH)_2_ nanosheet hybrid.

Figure 3 displays the scheme of the whole synthesis process. After the formation of α-Fe_2_O_3_ nanosheets, they were used as templates with CMC as the directing agent. The functionalized side groups on molecular chain of CMC are easy to combine with Ni^2+^ by electrostatic force to form polymer–inorganic composite, which can work as a template to ensure the anisotropic growth of the inorganic precursor and result in the formation of inorganic nanosheets^27^. The functionalized side groups of CMC can also enable the polymer–inorganic composite to be adsorbed onto the surface of α-Fe_2_O_3_ nanosheets due to the interaction between the functional groups (such as carboxyl and hydroxyl groups) and Fe^3+^. Under hydrothermal conditions, Ni(OH)_2_ nanosheets formed coating on the surface of α-Fe_2_O_3_. IR spectra of α-Fe_2_O_3_, Ni(OH)_2_, and Fe_2_O_3_@Ni(OH)_2_ are displayed in Figure S2 with the wavenumber ranging from 4000 to 300 cm^−1^. The sharp absorption peaks of around 3640 cm^−1^ and 533 cm^−1^ of both Ni(OH)_2_ and α-Fe_2_O_3_@Ni(OH)_2_ are related to stretching vibration and lattice vibration of hydroxyl respectively. Low wavenumber peaks around 534 cm^−1^ of α-Fe_2_O_3_, Ni(OH)_2_ and α-Fe_2_O_3_@Ni(OH)_2_ attribute to the lattice vibration of Ni-O, Fe-O, and nearly equal value indicates possible interaction between two compounds. Besides, the IR spectra also contain stretching (3404 cm^−1^) and bending vibration (1610 cm^−1^) of adsorbed water. No obvious extra peaks are observed in α-Fe_2_O_3_@Ni(OH)_2_, indicating no emergence of newly functional group, which illustrates possibly simple attachment of Ni(OH)_2_ onto the surface of α-Fe_2_O_3_ nanosheet.

**Figure 4:**
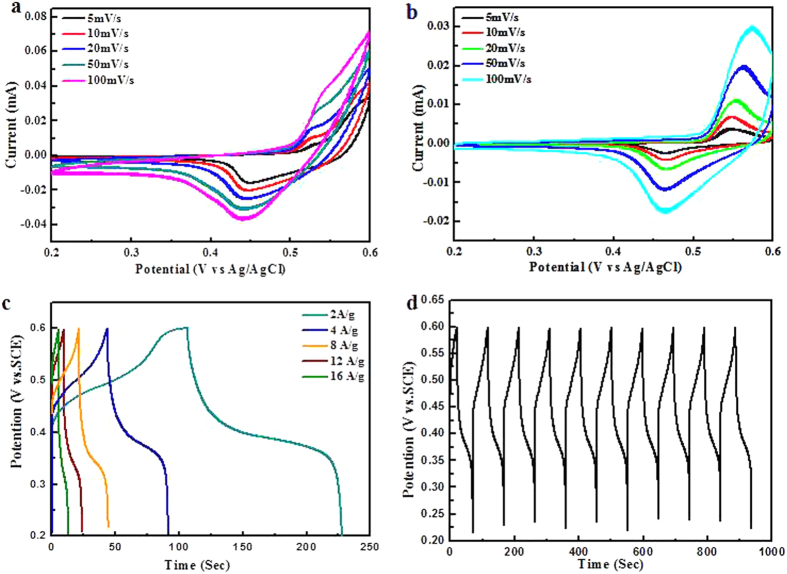
(**a**) CV curves of Ni(OH)_2_ nanosheets; (**b**) CV curves of α-Fe_2_O_3_@Ni(OH)_2_ nanosheet hybrids; (**c**) current charge/discharge curves of hybrid electrode at different current densities and (**d**) 10 cycles of charge-discharge curves of α-Fe_2_O_3_@Ni(OH)_2_ nanosheet hybrids.

The acquisition of the α-Fe_2_O_3_@Ni(OH)_2_ hybrid gives us opportunity to investigate the electrochemical performance of such novel nanosheet composites. In general, cyclic voltammetry (CV) is used to research the capacitive behavior and reversibility of an electrode material^5^. The electrochemical tests were carried out in a three-electrode system with a Pt wire counter electrode and an Ag/AgCl reference. Figure 4a,b show the CVs of the single Ni(OH)_2_ nanosheets and the α-Fe_2_O_3_@Ni(OH)_2_ nanosheet hybrid, which were conducted at the scan rates of 5, 10, 20, 50 and 100 mV/s with potential windows ranging from 0.2 to 0.6 V. Compared to the Ni(OH)_2_ electrode, although the CV peaks of α-Fe_2_O_3_@Ni(OH)_2_ hybrid electrode broaden, the two strong redox peaks still exist, which reveals the pseudocapacitance characteristics. The quasi symmetric characteristic of the redox peaks and cycles in the first few loops indicates the excellent reversibility of the Ni(OH)_2_ and hybrid electrodes. Figure 4c shows chronopotentiometry (CP) curves of the hybrid electrode within a potential window of 0.2–0.6 V at different current densities from 2 to 16 A/g. From the CP curves, it can be observed that the discharge time is longer than charge time at low current density, indicating that some undesirable reduction reaction may happen during the electrochemical process. It is also clearly observed from the CP curves that the each discharge curve contains two section: a rapid potential descent process and a slow potential decay process. The former represents a low internal resistance and the latter indicates the capacitive character of the electrode. Figure 4d shows the 10 charge-discharge cycles at a current density of 4 A/g, which indicates the superior reversible characteristics of hybrid electrode.

**Figure 5:**
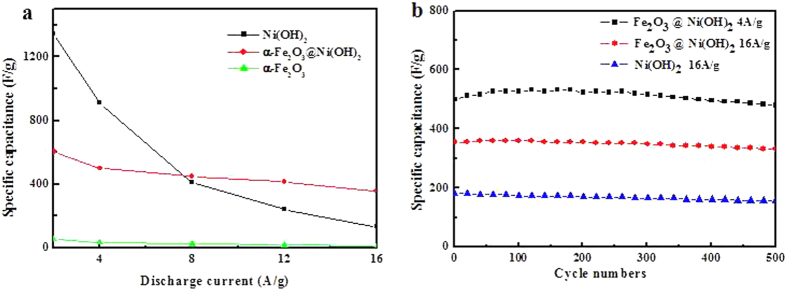
(**a**) Specific capacitances of α-Fe_2_O_3_ nanosheets, Ni(OH)_2_ nanosheets and α-Fe_2_O_3_@Ni(OH)_2_ nanosheet hybrid electrodes at different current densities; (**b**) cycling performances of the two electrodes.

To study the capacitance difference of Ni(OH)_2_ and hybrid electrodes, the CPs of Ni(OH)_2_ and α-Fe_2_O_3_ nanosheets were also carried out, which are displayed in Figure S3. The specific capacitance can be calculated from CPs according to the equation: C = (I_d_ × ΔT)/(ΔV × m), where C is the specific capacitance (F/g), I_d_ is discharge current(A), ΔT is discharge time (s), ΔV is the potential change (V), and m represents the mass of the active material within the electrode (g). Figure 5a shows the obvious specific capacitance difference of three electrodes at a current density of 2, 4, 8, 12 and 16 A/g, respectively, from which it can be clearly observed that α-Fe_2_O_3_@ Ni(OH)_2_ hybrid electrode has a smooth decrease of specific capacitance with increasing current density, compared with the sharp decline of single component of Ni(OH)_2_ electrode. The hybrid electrode exhibits higher specific capacitance than Ni(OH)_2_ electrode at high current densities, despite of lower capacitance than Ni(OH)_2_ at low current densities. As known, Ni(OH)_2_ is a promising pseudocapacitive material due to its high specific capacity but α-Fe_2_O_3_ is not an ideal material for supercapacitor electrode. At low current densities, the addition of α-Fe_2_O_3_ would no doubt sacrifice some capacity because of the low capacitance of α-Fe_2_O_3_. But in the other hand the better conductivity of α-Fe_2_O_3_ nanosheets would make the hybrid stand up the impact of large-current, which may attribute to the enhanced conductivity to support fast electron transport required by high rates^4^. EIS tests of α-Fe_2_O_3_, Ni(OH)_2_, and α-Fe_2_O_3_@Ni(OH)_2_ were carried out and the curves are shown in Figure S4a. From the semicircles in the EIS curves, it can be seen that the hybridation of α-Fe_2_O_3_ with Ni(OH)_2_ results in the decrease of charge-transfer resistance (Rct) comparing to that of single Ni(OH)_2_, indicating the more facile charge transfer ability of α-Fe_2_O_3_@Ni(OH)_2_ nanosheet hybrids, which may enhance the charge-discharge performance at high current densities. This is an exciting phenomenon that the rapid charging/discharging performance of Ni(OH)_2_ can be greatly improved by combining with the ultrathin ferric oxide. It is important for supercapacitor electrodes to keep capacitance extremely with low capacitance loss at long term circulations^28^. Therefore, cycling-life tests of the hybrid and the single component of Ni(OH)_2_ electrodes were carried out to discuss the behavior of capacitance decay. Figure 5b shows the cycling test of at a current density of 4 A/g and 16 A/g for the hybrid electrode and 16 A/g for the Ni(OH)_2_ electrode. The hybrid electrode presents good cycle stability with a extremely high capacity retention of 96.0% at a current density of 4 A/g and 93.3% at 16A/g after 500 cycles, confirming the good stability of hybrid electrode at both low rate and high rate. However, single component of Ni(OH)_2_ electrode displays a poor performance with a lower capacity retention of 81.8% at a current density of 16 A/g, which illustrates again that α-Fe_2_O_3_@ Ni(OH)_2_ nanosheet hybrid has an excellent performance of fast charging/discharging. At the same time, we also conducted the cycle performances of α-Fe_2_O_3_@Ni(OH)_2_ and Ni(OH)_2_ for 2000 cycles to research the long-term stability. As shown in Figure S4b after 2000 cycles, the hybrid electrode still has a higher specific capacitance than Ni(OH)_2_ electrode with good stability. The superior electrochemical performance of hybrid electrodes can be attributed to the synergistic effects of α-Fe_2_O_3_ and Ni(OH)_2_. α-Fe_2_O_3_ nanosheets not only provide abundant active sites for increased capacitance but also help Ni(OH)_2_ nanosheets stabilize nanostructure at high current densities. In this case, the superiority of 2D composite materials is reflected and the hybrid has potential to be developed as an outstanding supercapacitor electrode material. It is also highly expected to explore the supercapacitor devices in the future with α-Fe_2_O_3_@Ni(OH)_2_ nanosheets for high-performance supercapacitors with fast charging/discharging ability.

## Conclusions

In summary, α-Fe_2_O_3_ nanosheet@Ni(OH)_2_ nanosheet, a hybrid of two-dimensional ultrathin nanomaterial was synthesized and developed as electrode of supercapacitor through a simple and cost-effective approach. In such composites, α-Fe_2_O_3_ nanosheets serves as core and Ni(OH)_2_ nanosheets as shell, and this distinctive design effectively shortens the ion diffusion path and provides abundant active sites, meanwhile stabilizes the structure. The hybrid electrode presents a more smooth specific capacitance change than that of Ni(OH)_2_ electrode with the increase of current density. The electrode based on the hybrid composites shows excellent electrochemical performances at high current densities, including the increase of specific capacitance and enhancement of stability. The specific capacitance of α-Fe_2_O_3_ nanosheet@Ni(OH)_2_ nanosheet reached 356 F/g, and had a capacity retention of 93.3% at a current density of 16 A/g after 500 cycles, which was superior to the performance of Ni(OH)_2_ with a specific capacitance of 132 F/g and a capacity retention of 81.8%. The results illustrate that the hybrid electrode has a more excellent fast charging/discharging performance at high current densities than single component of Ni(OH)_2_. It is worthy to expect that the fabricated α-Fe_2_O_3_ nanosheet@Ni(OH)_2_ nanosheet composite architectures would be applied in high-performance supercapacitors with fast charging/discharging ability and high energy/power densities in the future.

## Methods

### Synthesis of α-Fe_2_O_3_@Ni(OH)_2_ nanosheet hybrid and Ni(OH)_2_ nanosheets

α-Fe_2_O_3_@Ni(OH)_2_ nanosheet hybrid material was prepared via a simple two step process. First, α-Fe_2_O_3_ nanosheets were synthesized by applying metal ions Al^3+^ as structure-directing agents, as described in our previous work^26^. Then α-Fe_2_O_3_ naonosheets were added to a Teflon-lined autoclave (40 mL) containing nickel acetate and carboxymethylcellulose aqueous solution under magnetic stirring. After 30 min of stirring, appropriate amount of ammonia solution (25%, analytically pure) was added to the autoclave for further 20 min of stirring. Then the mixture was sealed, transferred to oven, and kept at 80 °C for 12 h. The red precipitate was obtained by centrifugation (10 000 rpm, 1 min) and washed with deionized water and ethanol for 3 times, and dried in air. The synthesis of Ni(OH)_2_ nanosheet was same as α-Fe_2_O_3_@Ni(OH)_2_ hybrid except the addition of α-Fe_2_O_3_ nanosheets.

### Characterizations

Scanning electron microscopy (SEM) was performed on Hitachi S-4800 at 10 kV. Transmission electron microscopy (TEM) and high-resolution TEM (HRTEM) images were obtained by using a JEOL JEM-2100 transmission electron microscope operating at 200 kV. Powder X-ray diffraction (XRD) patterns were collected by using a Bruker D8 ADVANCE diffractometer with CuKα radiation (λ = 1.5418 Å).

### Electrochemical test

The working electrode was obtained by mixing the electroactive material, acetylene black, and polymer binder (polyvinylidene difluoride, PVDF) in a mass ratio of 75:15:10 with solvent (N-methylpyrrolidone, NMP). Then the homogeneous slurry was coated on the pre-treated nickel foam as the working electrode after stirring for one night, and dried at 50 °C for 12 h in a vacuum oven. Finally, the Ni foam with active materials was pressed with mass loading about 1.0 mg·cm^−2^ under 10 MPa for 40 seconds. Electrochemical measurements including cyclic voltammogram (CV) and chronopotentiometry (CP) were operated using a three-electrode system with a CHI 660 d electrochemical workstation in an electrolyte aqueous KOH electrolyte (1.0 M). A Pt wire was used as the counter electrode and a Ag/AgCl electrode was served as the reference electrode. In details, CV experiments were performed at various scan rates of 5, 10, 20, 50, and 100 mV/s. CP charge/discharge curves were obtained at various current densities of 2, 4, 8, 12 and 16A/g to evaluate the specific capacitance. A potential window in the range from 0.2 to 0.6 V was used during all measurements.

## Additional Information

**How to cite this article**: Jiang, H. *et al*. Hybrid α-Fe_2_O_3_@Ni(OH)_2_ nanosheet composite for high-rate-performance supercapacitor electrode. *Sci. Rep.* **6**, 31751; doi: 10.1038/srep31751 (2016).

## Supplementary Material

Supplementary Information
