# Supplementary material for: Hybrid α-Fe2O3@Ni(OH)2 nanosheet composite for high-rate-performance supercapacitor electrode
Source: Sci Rep. 2016 Aug 24;6:31751. doi: 10.1038/srep31751 (PMC4995458; doi:10.1038/srep31751)
Supplement: Supplementary Information [file srep31751-s1.doc]

**Supporting Information (SI)**

**Hybrid α-Fe_2_O_3_@Ni(OH)_2_ nanosheet composite for high-rate-performance supercapacitor electrode**

Hong Jiang,^1^ Haifeng Ma,^2^ Ying Jin,^2^ Lanfang Wang,^2^ Feng Gao,^1^,* Qingyi Lu^2^,*

**Figure S1.** SEM images of (a) single α-Fe_2_O_3_ nanosheets and (b) single Ni(OH)_2_ nanosheets.

**Figure S2.** IR spectra of single α-Fe_2_O_3_ nanosheets, single Ni(OH)_2_ nanosheets and α-Fe_2_O_3_@Ni(OH)_2_ nanosheet hybrids.

**Figure S3.** Charge/discharge curves of (a) Ni(OH)_2_ nanosheet and (b) α-Fe_2_O_3_ electrodes at different current densities

**Figure S4.** (a) Nyquist plots of EIS of α-Fe_2_O_3_, Ni(OH)_2_, and α-Fe_2_O_3_@Ni(OH)_2_ electrodes; (b) cycle performance of α-Fe_2_O_3_@Ni(OH)_2_ and Ni(OH)_2_ electrodes.
